# Utilization Intention of Community Pharmacy Service under the Dual Threats of Air Pollution and COVID-19 Epidemic: Moderating Effects of Knowledge and Attitude toward COVID-19

**DOI:** 10.3390/ijerph19063744

**Published:** 2022-03-21

**Authors:** Yueen-Mei Deng, Hong-Wei Wu, Hung-En Liao

**Affiliations:** 1 Department of Healthcare Management, Asia University, No.500, Lioufeng Rd., Taichung 41354, Taiwan; 2 Department of Pharmacy, Tajen University, No.2, Wexin Rd., Yampu 906, Taiwan; away7344@yahoo.com.tw

**Keywords:** air pollution, COVID-19, health belief model, pharmacy service, disease prevention

## Abstract

The utilization of pharmacy services in response to the threat of COVID-19 infection remains unclear in areas suffering from air pollution, and little is known regarding the effects of knowledge and attitude (KA) toward COVID-19 on this preventive behavior. This study aimed to explore how the residents perceived and reacted to the new threats of the epidemic and how KA may affect the correlation. Based on the health belief model (HBM), this research took the pharmacy service utilization (PSU) as an example to explain the preventive behavior. The samples were 375 respondents recruited from five districts near the industrial parks. T-test, ANOVA, and regression analyses of SPSS 22.0 were used to analyze the data. Test results show that self-efficacy was the strongest predictor, followed by the net perceived benefit. KA moderated the association of perceived threat and PSU intention. The levels of air pollution of a district may not be a good predictor for the preventive behavior against COVID-19.

## 1. Introduction

### 1.1. Reseach Background

The Coronavirus disease (COVID-19) pandemic has spread rapidly around the world with 291,241,130 confirmed cases and 5,458,356 deaths as of the date 4 January 2022 [1]. The pandemic had significantly altered every aspect of human life around the world. In Taiwan, the number of confirmed cases and deaths may be relatively small, 17,095 confirmed cases and 850 deaths, but the epidemic has noticeable influences on Taiwanese’s work and life behaviors.

Compared to the general coronavirus, COVID-19 attacks the human respiratory tract system more severely, and some cases will cause severe pneumonia and respiratory failure, and eventually lead to death. Prevailing treatments in response to the confirmed victim remain to support the human’s immune system to avoid the patient’s condition becoming severe after infection. Most severe cases of COVID-19 were found in the elderly or people with impaired or poor immune systems.

On the other hand, air pollution is one of the factors that are detrimental to human health, being accountable for 6.4 million deaths worldwide in 2015 [2,3], especially due to those particulate matters (PM) in the air with aerodynamic diameters ≤10 µm and ≤2.5 µm [4]. Both air pollution and COVID-19 are detrimental to humans’ respiratory health. Although have some argued that the lockdowns during the pandemic of COVID-19 have sharply mitigated the burden of air pollution around the world [5], residents in an area that had been exposed to high levels of PM have been proven to have a strong correlation with the deaths related to COVID-19 [4]. Compared to the general population, people who had suffered polluted air are apparently situated in a more detrimental condition with higher risks to their health, especially the respiratory system. Literature has evidenced that people who work or live in the neighborhood of risk source, e.g., epicenters, will perceive a higher risk of threats and will cause corresponding symptoms [6]. It will be interesting to know whether the threats of COVID-19 will be aggravated to the population who have long exposure to the risk of air pollution and accordingly affect their decisions in choosing preventive measures against COVID-19. Polluted air is one source of threats, and COVID-19 is another.

Although vaccination remains one of the primary measures to prevent infection of COVID-19, others measures such as physical distancing and personal hygiene, as well as nutrition supplementation for human immunity are also recommended [4].

Maintaining a sufficient level of required nutrition is essential to support the human immune system, however, decisions on the kinds and doses of each nutrition for each individual are complicated. It is wise to consult with the healthcare professionals such as pharmacists, nutritionists, physicians to gain optimal effects. To minimize the infection, people were advised to avoid crowded gatherings in a closed space, including medical institutes during the pandemic. Gaining advice from the pharmacists in a community pharmacy is a better alternative than visiting the physicians in a hospital in this special period.

Just as we are concerned about the service provided by a medical institute, pharmacy as an integrated part of the national healthcare system can provide a wide range of services to help people’s health needs, including how to prevent being infected by COVID-19.

The Central Epidemic Command Center (CEEC) of Taiwan hosts press conferences and provides news and informative data on COVID-19 daily as part of the nation’s anti-epidemic countermeasures. CEEC is the legal source of national level for collecting and disseminating the epidemic-associated information, including the number of confirmed cases, death toll, as well as the knowledge, anti-epidemic practices, and regulations regarding COVID-19. In the meantime, news and information of this kind are transmitted with multiple languages (including dialects and major foreign languages) to the public through various media 24 h a day. Under such continuous news bombardments, we can infer that the elderly, especially those aged at least 55, have been duly informed as to those most susceptible to severe illness when infected by COVID-19 in Taiwan.

The question was over how the people who are under such an environment of multiple threats will decide over the pharmacy service utilization (PSU) as a protective measure to prevent COVID-19. The purpose of this study is to explore how people who have already been exposed to the risk of air pollution in their daily life will react to the threats of being infected by COVID-19, and how this perception will affect their willingness to use pharmacy services as a measure to support their actions in lowering the risk of being infected and/or the possibility of becoming severe when being infected.

### 1.2. Literature Review

Poor air quality contains several pollutants in the air, such as PM_2.5_, PM_10_, O_3_, SO_2_ among others [7,8], in particular the particulate matters (PM). Adverse health effects of PM are mainly to induce pulmonary inflammation and consequently cause a higher risk of mortality and serious morbidity, especially for the population of the elderly, infants, and persons with chronic cardiopulmonary diseases or lung-related problems [7,8]. In Taiwan, the government adopted an Air Quality Index (AQI) to monitor the air quality, especially for those days with AQI > 100. AQI is composed of information of pollutants such as PM_2.5_, PM_10_, O_3_, SO_2_ among others that are harmful to human health. The air quality index (AQI) is composed of several sub-indicators of ozone (O_3_), fine suspended particulate matter (PM_2.5_, current standard at 15.0 μg/m^3^_/_year average, 35.0 μg/m^3^/24 H in Taiwan), suspended particulate matter (PM_10_, 50.0 μg/m^3^_/_year average, 100.0 μg/m^3^/24 H), carbon monoxide (CO, 35 ppm/1 H, 9 ppm/8 H), sulfur dioxide (SO_2_, 0.075 ppm/year average, 0.02 μg/m^3^/8 H) and nitrogen dioxide (NO_2_, 0.03 ppm/year average, 0.1 ppm/1 H), all of which are calculated according to their degree of impact on human health [9].

**Figure 1:**
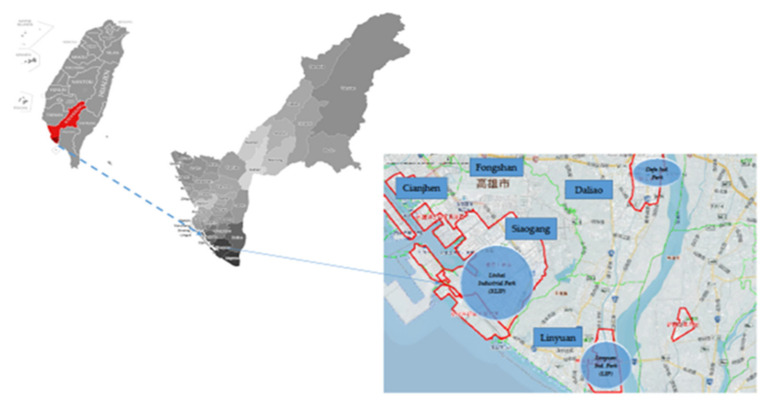
Industrial parks in southern Kaohsiung. Note: Ovals are industrial parks, rectangle names are administrative regions Source: Industrial land use and supply service, Industrial Development Bureau, MOEA, Taiwan, R.O.C. https://idbpark.moeaidb.gov.tw/ accessed on 9 March 2022.

In southern Taiwan, the most noted area of greatest concern for high air pollution risk is the neighborhood surrounding industrial parks, which covers Siaogang (Xiaogang), Linyuan, Cianjhen, Fongshan, and Daliao of Kaohsiung, as shown in Figure 1. Table 1 shows the number of days with AQI greater than 100 in each region [9]. The major source of air pollution that affects the air quality of the target districts are Kaohsiung Linhai Industrial Park (KLIP), Dafa Industrial Park (DIP), and Linyuan Industrial Park (LIP). KLIP accommodates 502 companies and more than 40,000 workers including giant steel, shipbuilding, cement, petroleum, and chemical companies in its 1582 hectares of land; and LIP covered 403.2 hectares of land to accompany 33 giant companies of plastic, chemical, and petroleum, whereas DIP accommodates metal processing companies in its area of 378.8 hectares. Siaogang is the home of KLIP, Daliao is for Dafa Industrial Park, and Linyuan is for LIP. Cianjhen district is not only a neighbor of these two industrial parks, it is also an ocean port for fishing and shipbuilding industries and the home of the previous Kaohsiung Export Processing Zone. Daliao and Fongshan are districts adjacent to both industrial parks in the same Kaohsiung Metropolitan area [10].

An AQI value of 100 or above indicates that the air quality is harmful to human health. Table 1 shows the number of days with AQI exceeding 100 in each region of this study. Despite that the days exceeding the standard are decreasing, all regions far exceed the national average, among which Siaogang is almost the highest on both of the days of AQI > 100 and the PM_2.5_ between years of 2018 and 2021, as shown in Table 1.

The community pharmacy plays important role in the anti-epidemic battle by providing easy-accessible and timely epidemic prevention supplies and medical equipment, such as small-package masks, over-the-counter (OTC) drugs, thermometers, alcohol, and other items to prevent infection.

In this special risky period of time, community pharmacy can be helpful in many ways [11], such as teaching and consulting appropriate hygiene techniques and methods, providing suggestions of nutrition intake, transmitting correct epidemic prevention knowledge, providing psychological (and emotional) support, assisting in preliminary tests for those suspicious customers such as body temperature, consulting travel history, etc., and help to monitor and report to the epidemic prevention agency for any suspicious cases [1].

Pharmacists and their community pharmacies are one of the best alternatives or substitutes other than physicians in the medical institutes for health promotion requirements, since pharmacists are the healthcare professionals who were trained to provide services of medication expertise and health promotion advice to the patients. Compared to other healthcare professionals, the pharmacists in the community pharmacy are particularly easy to reach by people. In Taiwan, 6603 community pharmacies around the country were included with a contract with the National Health Insurance Agency (NHIA) in the national healthcare system (there are an additional 1510 pharmacies which were not contracted with NHIA) [12].

Utilizing the pharmacy service is one of the best sources for health promotion decisions during the pandemic for at least several reasons as follows. Firstly, they are healthcare professionals with abundant healthcare knowledge; second, as a part of the national healthcare system, they can receive and disseminate timely information regarding national anti-epidemic measures; third, they store and provide anti-epidemic supplies (most of them are not allowed to be distributed in general channels) in a timely way; fourth, they are more familiar with the people and the environment of the area around the pharmacy than the other healthcare institutes; fifth, they have much easier access in terms of physical distance and business hours. In addition, community pharmacy that is contracted with the NHIB is particularly important for patients with chronic diseases to refill their choric prescriptions, especially in the epidemic season in Taiwan.

Theories that can be applied to explain health behavior are many; this study uses the health belief model (HBM) as a foundation to build the research framework of this study [13]. Compared to other theories on health behavior research, the logic behind the HBM is clear and straightforward; it is easier to communicate with participants and researchers than in the others [14].

The HBM was formulated with several constructs to predict a health behavior [15]. Perceived susceptibility and perceived severity in the HBM are sometimes combined and termed perceived threat; the former refers to a person’s perception about the possibility of getting a disease, and the latter refers to the feeling about the seriousness of the medical and social consequences of contracting such a disease. Perceived threat provides energy, whereas net perceived benefit (benefit minus barrier) provides a preferred path [16]. Perceived benefit refers to the benefits an action may bring to reduce the disease threat, whereas the perceived barrier refers to one’s belief of any negative outcomes accompanying the action. Self-efficacy was later included in 1988 as an additional predictor in HBM [17], which was defined as one’s conviction of successfully performed actions required for the expected outcomes [18]. HBM was then widely used in studies on disease precaution behaviors, such as disease prevention behaviors in varied contexts [19,20], including COVID-19 precautionary measures and associated behaviors [21]. In short, as a study on disease prevention behavior, HBM is the optimal theory for the current research context. Study results from the current research can be used to dialogue with previous studies as far as the precautionary behavior is concerned [22,23].

Psychological tension, as noted in many psychological studies [24,25,26], emerges when problems are aroused as a disruption to obstruct the expected routine of life. Tensions of this kind are problems that need to ease or be managed [27,28], and certain thinking and behaviors will be activated by the persons who perceived such tensions and problems [29]. Health is one of humans’ major concerns in almost every culture as it is the core for life survival, and the epidemic can be easily perceived as a threat and tension to human health when it breaks out. As a result, as the HBM studies asserted, corresponding behaviors in response to solving the threats will then be motivated and activated [6]. In the current research context, plenty of measures and instruments had been developed and suggested to prevent people from being infected by the pandemic of COVID-19. Vaccination, physical distancing, intensive hygiene, and strengthening human immunity are major streams of alternatives. The current research proposed that they are motivated to seek and evaluate the net benefits of each alternative including PSU in response to the threats of the epidemic.

The net benefit is the perceived benefits minus the perceived barriers of taking the recommended health action, which was termed as the “likelihood of action” as in previous studies [11,30]. Other than perceived threats and the likelihood of action of alternatives as predictors to the health behavior, the social learning theorist argued that vicarious learning is also an important role for individuals to activate a self-regulatory mechanism to learn a novel behavior or to change the current behavior without the trial and error process [31,32,33], as the links between cognition and behavior can be straight and direct [28,34,35].

The HBM-based studies added self-efficacy [17,36] as an additional variable and proved to have better explanatory capability [30]. Self-efficacy refers to an individual’s judgments of the capabilities s/he has that are required to gain the expected performances [32]. Self-efficacy is thus added to the HBM to cover the individual differences in the learning process, and as many HBM-based studies acclaimed, HBM with self-efficacy can better explain health promotion or disease prevention behaviors. We thus have a hypothesis based on the HBM as follows.

H1: The stronger the perceived threats, perceived likelihood of action, and self-efficacy, the stronger the intention of using the pharmacy service.

The current research was conducted to explain how the residents in the highly air-polluted area perceived the threats of COVID-19. Since residents of such areas may have been alerted to the impaired function of the respiratory system due to poor air quality, it is logical for these people to seek alternatives for health protection, such as revitalizing the immune system through PSU. As a result, the levels of perceived threats of COVID-19, perceived likelihood of actions, and PSU intention can be higher than in those people who were not exposed to air pollution. Since Siaogang exposed the most to air pollution among the five districts of the research, we therefore proposed a hypothesis as follows.

H2: Perceived threats, perceived likelihood of action, and pharmacy service utilization intention will be higher for people in Siaogang than those of other districts.

According to HBM, the threat of COVID-19 infection, and the benefits and barriers of specific measures in response to the threats are the main predictors of such behavior. This study believes that HBM predictors are originated or affected by personal subjective opinions. As bounded rationality theory suggested, personal rationality is limited by one’s own levels of knowledge. In other words, the individual’s knowledge and attitude (KA) regarding COVID-19 will affect the individual’s cognition of disease threats, the perceived benefits of the focus measure against the COVID-19, and the actions to be taken. Therefore, unlike some literature that suggested examining possible mediation and moderation between the core components of the HBM than exploring direct effects [37], the current research attempts to include KA toward the threats as an additional factor in the model to expand our understanding in a different perspective. Since knowledge and attitude basically act as the foundation to motivate and shape human behavior, we thus proposed a hypothesis as follows.

H3: Knowledge and attitude regarding COVID-19 moderate the relationship between perceived threat, net likelihood of action, self-efficacy, and pharmacy service utilization intention.

By accomplishing the purpose of the research, we wish to shed more light on how the knowledge and attitude regarding COVID-19 will affect the known relationship of HBM, in particular for the residents of highly air-polluted areas. In the meantime, it will also provide better insight into how people will react to protect their health when additional threats from the environment emerge.

## 2. Materials and Methods

### 2.1. Materials

Research material is the data gathered from customers who purchase health products from pharmacies in the five districts in the neighborhood of two major industrial parks of heavy industries in Kaohsiung, Taiwan. The survey was conducted in the month of December 2021. Participants responded to a recruiting poster as a volunteer to this study. Participants were asked to complete a paper questionnaire right after completing a transaction with the pharmacy. A gift worth around 100 NTD was then provided as a return of survey completion. Out of 400 participants, 25 responses were invalid because of one or more variables were left blank or due to conflicting demographic factors (e.g., healthcare professional yet high school degree, which violates statuary requirement), and 375 valid responses were included for analysis.

The research project was approved by the Research Ethics Committee of Jen-Ai Hospital in Taichung, Taiwan with IRB Case No. 110-88 (18 February 2021) before the study was implemented.

### 2.2. Measurements

A structural questionnaire was composed of three parts, the first part aimed to measure the respondent’s perceived threats regarding COVID-19 and perceived benefits and barriers of PSU. The second part is to measure the respondent’s KA levels toward COVID-19. The third part is used to collect socio-demographic information.

According to the HBM, the five constructs included are susceptibility, severity, benefit, barrier, and self-efficacy. Susceptibility is a construct to measure one’s perception toward the possibility of contracting a certain disease or health problem. As far as this study is concerned, it is used to measure how likely the subject perceives themselves to be infected by the COVID-19 virus. Eight items are included in this scale, such as “How likely will you be infected by COVID-19, because of working with your clients?”. Possible sources of infection are family members, colleagues, clients, journeys, public transportation, restaurant, closed rooms, and other high prevalent countries or districts. A five-point scale is used, 1 for extremely unlikely, and 5 for extremely likely. Reliability is acceptable at the level of Cronbach’s α = 0.900.

The construct of severity is to measure how intolerable the outcomes that may be caused by certain diseases or health problems are, such as COVID-19 in this study. There are 8 items covering major health problems caused by COVID-19, such as loss of smell and taste, headache, cough, wheezing or difficulty breathing, diarrhea, liver and kidney dysfunction, heart problems, pneumonia, and mental health problems. A five-point scale is used, 1 for highly tolerable, and 5 for highly intolerable. Reliability is at the level of Cronbach’s α = 0.885.

The perceived threat of COVID-19 is a construct to measure the possibility of being infected and the severity of the outcomes when infected. In this study, we calculate that the perceived threat as a product of the square root of perceived susceptibility multiplies the perceived severity, shown as Equation (1). The equation denotes that the higher the susceptibility and the severity of being infected, and the stronger it is to perceive the threat of COVID-19.

(1) $$\mathrm{COVID}\text{-}19\;threat=\sqrt[{2}]{\left(suceptibility\;X\;severity\right)}$$

Perceived benefit refers to the perception of the positive outcomes that result from a specific action. In health behavioral studies, this term is frequently used to explain or predict one’s motives of conducting behavior of disease prevention or health promotion [18]. In this study, we adopted the term to describe the benefits of PSU that the respondents perceive in helping them strengthen their prevention efficacy against COVID-19. This study used eight items, such as “Pharmacy can be beneficial in advising optimal healthy products”, to measure the concept of perceived benefit. Reliability is acceptable at the level of Cronbach’s α = 0.923.

In contrast to the benefit, a barrier means something that impedes or separates the individual from the expected behavior or outcome. Perceived barrier thus refers to one’s personal judgment of the degree of difficulty of factors that can impede the accomplishment of specific health behavior [38]. This study used seven items, such as “Prices of PSU is too costly”, to measure the concept of the perceived barrier. Reliability is acceptable at the level of Cronbach’s α = 0.845.

We use the term likelihood of action of PSU intention to reflect the balance of perception of one’s evaluation on PSU. The value of such balance is calculated as the balance of benefit and barrier. We thus subtract the average value of barriers from the average value of perceived benefits, as shown Equation (2), and take the net value as the net perceived value for further analysis. This means the more positive the balance, the stronger the confidence the individual may possess for the focal behavior.

*Likelihood of action = Benefit average − Barrier average* (2)

There are five items in this construct to measure the respondent’s PSU intention. One example for this scale is “I will make use of pharmacy service as frequently as possible to gain healthcare advice”. A five-point scale is used, 1 for totally disagree, and 5 for totally agree. Reliability is at the level of Cronbach’s α = 0.938.

Self-efficacy is a term that refers to one’s belief incapacity to conduct behaviors necessary to gain specific outcomes [18,32,38]. Levels of self-efficacy may indicate one’s confidence in the ability to exert control over one’s motivation, behavior, and social environment. Five items, such as “No matter how high of the price, I will regularly utilize pharmacy service to protect my health”, are included to measure the respondent’s self-efficacy. A five-point scale is used, 1 for totally disagree, and 5 for totally agree. Reliability is at a level of Cronbach’s α = 0.925.

Mainland China is the region where the COVID-19 virus was first identified, its counter-measures and research on COVID-19 also started the earliest, and a scale was then developed with the samples from the highly populated and infected area in China by Peng and colleagues (2020) to capture the resident’s knowledge, attitude, and practice (KAP) regarding COVID-19 [39]. The instrument was then widely adopted as a basic tool for KAP research in many countries and regions, such as Malaysia [40], Indonesia [41], Japan [42], Egypt [43], Turkey [44], Saudi Arabia [45], Spain [46], and Italy [47] among others. The instrument contained five items for each KAP variable. Since China and Taiwan are culturally similar to each other, we adopted two of the scales to measure the respondent’s knowledge and attitude (KA) in this research. Five questions with three choices each are used to measure the knowledge. Questions such as “What type of infectious disease is COVID-19?” with three choices are presented with each question. For each correct answer, 1 point is calculated. Scale to measure attitude contains five questions as well with the same grading structure. Question for attitude, such as “Are you scared by the human-to-human transmission of COVID-19?”, with two to three options were presented to gain responses.

### 2.3. Data Analysis

Descriptive analysis was used to describe the sample profile; *t*-tests and one-way ANOVA were used to examine the differences in average scores of each variable for each demographic factor. Multiple regressions were applied to test H1 for the effects of the independent variables of perceived threats, net perceived benefit, and self-efficacy on PSU intention, to test whether the most polluted area of Siaogang will have higher threat, net benefit perception, and PSU intention than other areas, and to test H3 for the moderating effects of KA on the links with PSU. The analytic techniques were applied using the SPSS 22 software package (Armonk, NY: IBM Corp. Sourced from TriStar, Kaohsiung City, Taiwan). All significance levels were set at *p* ≤ 0.05.

## 3. Results

### 3.1. Subjects Profile

There were 375 valid responses included in the current study, of which 58.13% were female, 64.27% married. More than 94% aged under 60 years old, 82% hold at least a college degree, one third (32.53%) of respondents were home keepers that include retired and jobless persons, and around 85% earned no more than 60 K (around 2000 USD) per month, detailed as shown in Table 2.

### 3.2. Analyses of Variables

Perceived severity received the highest average score at 4.12 ± 0.81 among all predictors at a 5-point scale, followed by perceived benefit (3.74 ± 0.65) and self-efficacy (3.65 ± 0.78). Compared to perceived benefit as one of the top-tier variables, the perceived barrier has the lowest score at 2.90 ± 0.74 indicating a general incline to the likelihood of action. The average scores for knowledge regarding COVID 19 are 3.72 ± 0.86 and 3.58 ± 0.89 for attitude.

As far as the socio-demographic factors are concerned, age and resident area appear to be the most significant factors in segmenting the values of variables, followed by income levels, jobs, and educational levels.

No significant differences are found for gender and marital status. The levels of education bring minor effects on the predictors. Only the perceived barrier (*p* = 0.018 < 0.05) has a statistically significant difference in terms of educational levels, and the respondents who are educated as “high school and under” are significantly higher than those educated with “bachelor” degrees.

The difference in the type of jobs, as other demographic factors do, has some effects on the values of each variable [48]. The “state employee” perceived higher susceptibility than those of “office workers” employed by private enterprises and “home-keeper” including retired (*p* = 0.010 < 0.05). In the perceived severity, the score of “healthcare” professionals is significantly higher than those for private business employees and self-employed businesses (*p* < 0.001 < 0.05), as shown in Table 2.

As to the different age groups of respondents, differences in some variables can be also found. In the perceived severity, respondents aged “41–50” and “51–60” are higher than those aged “under 30” (*p* = 0.019 < 0.05). In the perceived barrier, respondents aged 30 and under perceived more difficulties than their older counterparts (aged 41–60) (*p* = 0.038 < 0.05). In terms of self-efficacy, the older ages of the “51–60” group are higher than the “31–40” ages (*p* = 0.002 < 0.05).

In income levels, the respondents with a higher income of at least 60 K significantly perceived more benefit of (*p* < 0.001 < 0.05), higher self-efficacy (*p* = 0.008 < 0.05), and are more inclined to PSU intention (*p* = 0.004 < 0.05) than those respondents in the lower three levels (i.e., less than 50 K). For similar reasons to those in the age factor, lower income may act as an obstacle preventing them from PSU intention, as shown in Table 2.

On the other hand, significant differences are found in the perceived susceptibility, self-efficacy, and personal knowledge and attitude regarding COVID-19. Siaogang has the highest score of perceived susceptibility of being a victim of the COVID-19. Compared to the average score at 3.36 ± 0.81 on a 5-point scale for the perceived susceptibility, residents here are much more suspicious of being infected (3.84 ± 0.67). On the contrary, they have the lowest score of self-efficacy at 3.41 ± 0.79, significantly lower than those for Daliao (3.80 ± 0.60), Fongshan (3.99 ± 0.80), and the average of this research (3.65 ± 0.78). Compared to other districts of this research, Cianjhen is the district whose residents have the second-lowest score of self-efficacy (3.49 ± 0.77) of PSU intention, as shown in Table 3.

### 3.3. Associations among Variables

Regression analyses, after controlling for socio-demographic factors, indicated that perceived threat (susceptibility and severity) (*β =* 0.189, *p <* 0.001), likelihood of action (*β =* 0.275, *p <* 0.001), and self-efficacy (*β =* 0.441, *p <* 0.001) are significantly effective in predicting the PSU intention with 39.0% of variance explained (*R^2^* = 0.390), among which the self-efficacy is the strongest predictor, as shown in the Table 4. H1 of the current research is thus supported and confirmed that the stronger the perceived threats, perceived likelihood of action, and self-efficacy, the higher the intention of PSU.

A regression analysis with controlling the socio-demographic factors is performed to reveal the correlations of perceived threats, net perceived benefit, and PSU intention with each district in question. Variances explained by the area are 12.9%,7.0%, and 5.4% for the perceived threat, the likelihood of action, and the PSU as dependent variables, respectively. As H2 hypothesized, the perceived threat of residents in Siaogang is stronger than Cianjhen (*β* = −0.257, *p* < 0.001), Daliao (*β* = −0.262, *p* < 0.001), Fongshan (*β* = −0.301, *p* < 0.001), and Linyuan (*β* = −0.307, *p* < 0.001), as shown in Table 5. Daliao (*β* = 0.069, *p* = 0.240, non-significant) has stronger coefficient in the likelihood of action than Cianjhen (*β* = −0.128, *p* = 0.041), Fongshan (*β* = −0.018, *p* = 0.759, n. s.), and Linyuan (*β* = −0.012, *p* < 0.836, n. s.). The test also shows that Daliao (*β* = 0.034, *p* = 0.565, non-significant) also has stronger coefficient in PSU intention than that for Cianjhen (*β* = −0.131, *p* = 0.039), Fongshan (*β* = 0.029, *p* = 0.633, n. s.), and Linyuan (*β* = −0.023, *p* < 0.691, n. s.). H2 hypothesized that perceived threats, the likelihood of action, and pharmacy service utilization intention will be higher for people in Siaogang than those of other districts. Test results in Table 5 indicate that only the perceived threat of residents in Siaogang is significantly higher than that in other areas, and the remaining two variables of the likelihood of action and PSU intention are not supported. Therefore, H2 is partially supported.

**Figure 2:**
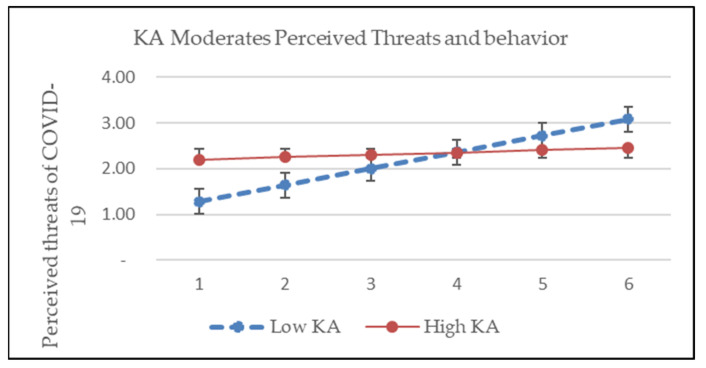
KA moderates perceived threats of COVID-19.

**Figure 3:**
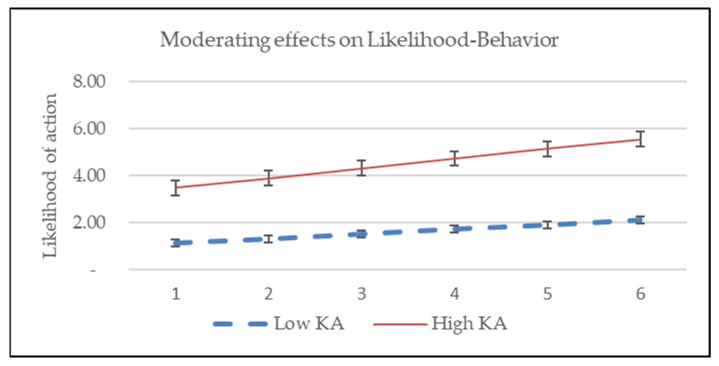
KA has no moderating effects on likelihood of action and intention.

**Figure 4:**
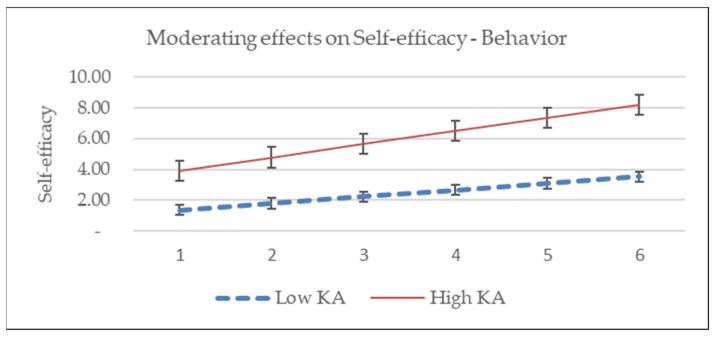
KA has no moderating effects on self-efficacy and intention.

This research hypothesized that levels of knowledge and attitude regarding COVID-19 will moderate the associations of predictors and PSU intention. This means that the coefficient of each variable as well as the association between dependent and independent variables are fluctuating along with the levels of knowledge and attitude regarding COVID-19. We test the moderating effects of KA on the associations between PSU intention and perceived threats, likelihood of action, and self-efficacy. The significance of the interaction will reveal whether there is a moderating effect between the independent and dependent variables [49]. Test results indicate that the interaction of perceived threat and KA is significant (*p* = 0.042), and interactions of KA and the likelihood (*p* = 0.635) and self-efficacy (*p* = 0.300) were not significant, as shown in Table 6. This means the levels of KA moderate the association between perceived threat and PSU intention, and not work the same for the links of PSU-Likelihood and PSU-Self-efficacy, as shown in Figure 2, Figure 3 and Figure 4. H3 of the current research is partially supported.

## 4. Discussion

COVID-19 overwhelmingly changed human behavior and social life. Before certain innovative treatments were developed and confirmed to be effective, the current knowledge and techniques that humans had learned and practiced for health promotion and disease prevention will still be the best practice for us to use against COVID-19. Although Taiwan is now able to gain sufficient vaccines from foreign sources and local biotech suppliers, the threats of infection remain high because virus variants are emerging. Vaccination, a healthy diet, a healthy lifestyle, and sufficient nutrition remain the optimal practices to deal with the threats of COVID-19. The community pharmacy in the neighborhood can serve as a reliable station to consolidate and supply the correct information and to provide advice and preventive instruments to support the community residents in this anti-epidemic battle.

### 4.1. Variations of Perceived Threats and Benefits

According to the HBM model, an individual’s scores on the four variables can predict a corresponding behavior. In short, susceptibility and severity are positively associated with a preventive measure and the net perceived benefit of such measure. In the meta-analysis studies, the barriers, benefits, susceptibility, and susceptibility were good predictors of health behaviors, of which benefits and barriers are the most consistent predictors [13,14,15,50].

Residents in the area under heavy threats of air pollution perceived lower susceptibility in average scores than the perceived severity. This means respondents are more concerned about the severity of infection than the possibility of being infected. This may infer that residents are negligent about the possibility of COVID-19 infection, but are very concerned about the severity of the outcomes in the case that they were infected [51]. Although the efficacies of perceived susceptibility and severity in explaining health behavior had been confirmed, a consensus was not reached on which of the susceptibility or the severity will be the stronger predictor in the health behavior studies, which may be con-text-specific [13,14,15,50].

No matter which of the vulnerability or the severity is stronger, both of them are valid predictors to examine the subject’s perceived threat of diseases or health risks. It is a given that the magnitude of susceptibility and severity perceived by each respondent may not synchronize with each other. Respondents with the same degree of susceptibility may not perceive the same severity and vice versa. To get closer to the real world, we use the product of susceptibility and severity as a whole of perceived threat to predict the dependent variable. The perceived benefit was confirmed as the strongest predictor in a meta-analysis study [50], as the current study indicated that the likelihood of action is the stronger predictor than perceived threats [52]. In short, the current study revealed rather consistent results with previous studies that show that the perceived threat (susceptibility and severity) and the likelihood of action (benefit and barrier) are significant predictors of the PSU intention.

### 4.2. District and Socioeconomic Factors of Samples from High Air Pollution Regions

Literature had confirmed that air pollution is inclined to cause cardiovascular diseases (CVD) and the respiratory system for vulnerable groups, in particular the racial/ethnic minorities, women, the elderly, smokers, diabetics, and those with prior heart diseases [53].

### 4.3. Area

As far as the residential area is concerned, ANOVA analyses in this research indicate that no significant differences are found in the perceived benefit and barrier, and PSU intention across different districts. This implies that no matter the distance from the source of pollution, the residents around these two industrial parks agree with the benefits of PSU intention in preventing COVID-19, and have no problem in accessing such products. High in perceived benefit and low in perceived barrier predict a similar direction of the likelihood of action across the five districts in this investigation.

Geographical distance to the source of air pollution determines the severity of being influenced by such bad air, so too their perceived susceptibility. Perceptions regarding predictors and intention are varied along with the geographical distances of each district to the source of pollution. Three of the five districts in this study are homes of industrial parks, and the other two are geographical adjacencies. As people whose residence near the center of the epidemic perceived a higher risk of being affected and consequently higher depression and anxiety [11], it is logical for the respondents of Siaogang, the home of the biggest cluster of heavy industries of KLIP, to perceive the highest threat of infection. People in the highly air polluted area may have developed a pearl of wisdom and practices to accommodate such harsh environments. Compared to other districts, Fongshan is a more urban area. It was previously the capital of the Kaohsiung county before it was merged into the current Kaohsiung Metropolitan City, and currently one of the administrative centers of the city. Interesting to note is that the perceived threat of being infected by COVID-19 for residents in Fongshan is stronger than those in Linyuan only. Similar to previous studies, a significant determinant of fear of health risks was not the outcomes of air pollution on health problems, rural or urban residence, but the distance to the source of risk as well as other demographic factors [54].

As far as knowledge and attitude are concerned, respondents from Siaogang appear to have higher scores of knowledge and attitude than those from Fongshan and Linyuan. Ironically, the Linyuan district was generally viewed as a hazardous area of air pollution of chemical and petroleum manufacturing.

PSU intention is high in this study and there is no significant differences among the five districts in different geographic distances which is consistent with previous studies [55]. Worthy to note is the significantly lower values of PSU intention of Cianjhen district, which perceives the second-highest threat of COVID-19 infection in this research. Cianjhen is the home of Kaohsiung Export Processing Zone (KEPZ), the first EPZ in the world that was established in 1966. Residents in this district may have accessed and been affected by the foreign cultures much earlier than the other areas since barely all firms in KEPZ are foreign equity. This may mean that Cianjhen is a different research setting from other districts in this research. Age. As far as the perceived threat of COVID is concerned, the current research found that the perceived susceptibility of older ages is not significantly different from other age groups, similar to a previous study using data from 27 countries [56]. However, this research has also found that the oldest people perceived significantly higher severity than those younger, which is similar to another study that concluded that the older Americans were more worried about infection [57]. A possible reason for such higher scores of the severity of these two groups with older ages, “41–50” and “51–60”, is that they may be influenced by the memories of the threat of earlier worldwide pandemic of SARS that prevailed in 2003.

Self-efficacy refers to the respondent’s judgment on the balance of resources and capabilities one owned to offset the requirements of achieving the target goal. The older people had witnessed the threat of SARS 2003 on one hand, and are more financially resourceful on another. Therefore, it is logical as the test results had shown. In addition, a younger age means an entry-level of jobs with lower income and consequently a lower portion of discretional income. The physical status of the young is always healthy with a lower possibility of vitamin deficiency and health problems, and thus they have stronger confidence in the health of their immune system.

Worthy to note is that the females in the middle ages are generally the main health decision-makers in the family. It is often the age of being a major caregiver and behavior influencer of children at school age. Mothers’ knowledge, attitude, practice regarding health promotion and disease prevention will, highly likely, shape the children’s health-associated behavior in their future life.

Studies may differ in categorizing age groups, e.g., 70 years old in the previous studies [56] and 61 years old in this research are classified as the elderly, and thus conclusions can be somehow diverse, but the fear of infection is there and rises in line with age increase. Although some variations may exist across studies, age remains one of the significant predictors of major physical and mental health problems. No matter how the studies differ in defining the elderly, older people are apparently more vulnerable than their younger counterparts and deserve special care.

### 4.4. Gender

Although women are the majority in the frontlines of healthcare work [58] and are the primary healthcare givers to the family, and the jobs of this kind are usually exposed to a riskier context with a higher possibility of contagion, a recent study based on several national statistics reported that male and female have relatively even distribution in infection rate, but the male had a higher share of the number of deaths [58,59]. Other than biological or physiological factors (such as sex hormones and sex differences in disease progress etc.) and risky health behavior (such as smoking and binge-drinking) [59], experiences gained from daily works may help women develop a protection motivation along with a set of anti-epidemic measures to cope with the threats [60]. They are more likely to be worried about COVID infection [57,61], and that in turn prevents them from being infected and becoming severely ill. This study indicated no significant differences between males and females in terms of perceived susceptibility, which is inconsistent with the literature that suggested this.

### 4.5. Education

People with low education levels feel relatively obstructed in the access to PSU intention. Lower levels of education may mean a higher possibility of being convinced by mass media and the environment.

### 4.6. Knowledge and Attitude as Moderator

Moderator is one of the interesting issues in the studies based on HBM, some variables had been raised as effective moderators such as prevention vs. treatment, drug outcome vs. non-drug outcome, age, sexual orientation, and time intervals among others [36,62,63]. This research indicates that the levels of knowledge and attitude regarding COVID-19 moderates the relationship between perceived threats and PSU intention, and not for the relationship of the link between net perceived benefit and self-efficacy.

Knowledge and attitude can shape human behavior through direct or indirect mediating or moderating, or both effects. This research revealed that the effects of perceived threats were shaped by the levels of knowledge and attitude. The more knowledgeable and positive the attitude of a person, the stronger the association of the perceived threats and PSU intention. The moderating effects were not significant for the relationships between net benefit and self-efficacy on the PSU behavior, respectively. Since the effects of perceived threats can be magnified by knowledge and attitude, it implies that supplying timely and sufficient information on COVID-19 to the public or targeted segments is important for policymakers and health product marketers.

KA levels in this research are varied from one to another in terms of education, income levels, jobs, and area, and there are no significant differences in sex, age, and marital status in this research. KA is inclined to be optimal for the highly educated population who owned bachelor or master degrees than those who received high school or lower education. Education is important for effective communication, yet a high school education will be enough for people to apprehend the message regarding COVID-19, especially when the messages were encoded using varied languages (or dialects).

Information regarding anti-epidemic shall be made clear and easy to access by the public in a timely manner to foster the nation’s sufficient and effective knowledge and attitude toward the epidemic, and accordingly, establish proper personal protection strategies. The government shall not manipulate the ways and quantity of information flow but leave it open to avoid panic and confusion and ensure the nation’s health.

### 4.7. Telepharmacy

As far as the barrier to PSU is concerned, one of the possible solutions is the adoption of telepharmacy, which was developed by taking advantage of the advanced information and communication technology (ICT), such as telephone, video call or conferencing, and associated software and processes [64]. Telepharmacy refers to a form of pharmaceutical services delivery to patients who are not able to meet pharmacists in person [65]. Service of this kind can be particularly good for the patients who are geographically or physically isolated, and or for those who cannot access the accredited service sites to counter certain levels of barriers [66], and accordingly increase the accessibility of pharmaceutical care [67]. Telepharmacy provides physical distancing, as is required to minimize the diffusion of COVID-19 infection, especially for those patients with respiratory problems. In the meantime, both pharmacy service providers and receivers can share the benefits of cost and time-saving. Taiwan started to adopt telemedicine in 1995, and later to include telepharmacy in 2007 in some selected hospitals, however it never been popular since then. Major constraints of the diffusion of the system in Taiwan just like those in other countries are legislative regulation, privacy, and financial cost of telepharmacy facilities, as well as the insufficient ICT literacy of pharmacists [66]. Given that telepharmacy can also significantly ease the inequality of medicine service distribution, shortage of pharmacists, and timely health service to the people that related to the national health policy, the government shall actively get involved to remove such diffusion barriers.

## 5. Conclusions, Limitations, and Future Research

### 5.1. Conlusions

Self-efficacy is the strongest predictor of PSU intention, followed by net perceived benefits, and perceived threats. Levels of knowledge and attitude can moderate the effects of perceived threats on supplementation, and not for the other associations in the model.

The area is not a significant variable in differentiating values of dependent and independent variables in the model, but other socioeconomic factors, such as age, income, and occupation brought certain effects. Whether the area was already heavily exposed to air pollution may not be a good indicator to predict the prevention behavior against COVID-19.

### 5.2. Limitation of the Study

The first and the main limitation of this study is that the samples targeted the residents in the air-polluted area, which inevitably excluded the population that does not reside in these areas. This may prevent us from comparing the test results with those in non-or less air-polluted areas. The second limitation is that the samples were not randomly collected but in a convenient way from the pharmacy stores within the target area. This means the generalizability of the test results may not be feasible for the national population and shall be carefully interpreted. However, it will be a valuable reference for those contexts under the same problem of air pollution. The third limitation is that the study was conducted in a special time period when the locally confirmed cases of infection had been significantly decreased to a fractional number per day, and some variants such as Omicron had been found to start to break through the vaccine line in Taiwan. It is almost impossible to forecast how the epidemic will progress with the emergence of variants and the outcomes of anti-epidemic measures, as well as how these will affect human life in the future. The context of this particular research may not be the same as others will be in different environments. The scenario people experience now is an environment featured with a sufficient supply of vaccines and healthcare resources, confirmed local cases are well-controlled, and few restrictions of social interactions and business transactions, which are quite different from those in other countries. Test results of this research shall only be applicable to those studies in a similar context with new threats emerging to an already compromised area.

### 5.3. Future Research Directions

Since air pollution is not a good indicator to explain the prevention behavior of residents in an such area that has been exposed to such pollution for a long time, we suggested making a further study on those who newly immigrated to the area, as well as on the areas whose residents are new to the air pollution. Other studies that focus on other possible factors that can neutralize the negative impacts of bad air on prevention, such as that originating from the inequalities of economic, informative, and educational factors, and that stemmed from the disparities of health literacy and access of information and healthcare resources, deserve to be conducted. A study of that kind was not to provide instruction for governments to evade environmental protection responsibility, but to remind the local community not to neglect the hazards of air pollution.

Other than the socio-demographic factors, we have no evidence to examine whether the possible effects of other factors exist in differentiating the efficacies of predictors and the intention of PSU. This may include those minorities who have less access to the healthcare resources, those who stayed in an area where at the time featured with either economic, political, or social turbulence, by which they were easily ignored by the social system. These deserve further investigation, for the epidemic of COVID-19 is ubiquitous on this planet.

## Figures and Tables

**Table 1:** AQI of focus areas in the years between 2018–2021. Source: Data are drawn from the statistics of the Environment protection administration, Executive Yuan, Taiwan, R.O.C. AQI, air quality index; Avg., National average; A, Days, AQI > 100; B, Days, PM2.5 > standard.

Year	2018		2019		2020		2021	
Station/days	A	B	A	B	A	B	A	B
Avg.	141	91	43	19	32	10	33	18
A1 Cianjhen	210	209	98	59	74	32	64	45
A2 Daliao	243	170	57	57	30	30	73	56
A3 Fongshan	217	212	46	46	20	20	54	54
A4 Linyuan	245	71	67	36	53	18	102	41
A5 Siaogang	249	215	98	47	79	27	57	47

**Table 2:** Demographic profile.

Factors	Category	*n*	%
Gender	Male	157	41.87
	Female	218	58.13
Age	<30	73	19.47
	31–40	114	30.40
	41–50	101	26.93
	51–60	65	17.33
	>61	22	5.87
Marriage	Married	241	64.27
	Single	134	35.73
Education	<High School	66	17.60
	College	244	65.07
	Master’s & up	65	17.33
Occupation	Office workers	109	29.07
	State employee	38	10.13
	Self-business	56	14.93
	Healthcare	50	13.33
	Home keeping	122	32.53
Income	30 K or lower	45	12.00
	31–40 K	114	30.40
	41–50 K	104	27.73
	51–60 K	56	14.93
	61 K and up	56	14.93
District	Cianjhen	55	14.67
	Daliao	61	16.27
	Fongshan	67	17.87
	Linyuan	75	20.00
	Siaogang	117	31.20

*n* = 375.

**Table 3:** Summary of the results of the *t*-tests and ANOVA. M = mean; SD = standard deviation; n. s. = non-significant; Sus. = perceived susceptibility; Sev. = perceived severity; Ben. = perceived benefit; Bar. = perceived barrier; Int. = PSU intention; SE = Self-efficacy; Know. = knowledge; Att. = Attitude; * *p* < 0.05, ** *p* < 0.01, *** *p* < 0.001. ^a^ 1. < 30, 2. 31–40, 3. 41–50, 4. 51–60, 5. 61 & up; ^b^ 1. High school and lower, 2. Bachelor, 3. Masters and up; ^c^ 1. < 30 K, 2. 31–40 K, 3. 41–50 K, 4. 51–60 K, 5. 60 K & up; ^d^ 1. Office worker, 2. State employee, 3. Self-business, 4. Healthcare, 5. Home-keeping; ^e^ 1. Cianjhen, 2. Daliao, 3. Fongshan, 4. Linyuan, 5. Siaogang.

Var.	M	SD	Sex	Mar.	Age ^a^	Edu ^b^	Income ^c^	Job ^d^	Area ^e^
Sus.	3.36	0.81	n. s.	n. s.	n. s.	n. s.	n. s.	2 > 1,5 *	5 > 1,2,3,4 ***
Sev.	4.12	0.68	n. s.	n. s.	3,4 > 1	n. s.	n. s.	4 > 1,3,5 ***	n. s.
Ben.	3.74	0.65	n. s.	n. s.	n. s.	n. s.	5 > 1,2 ***	n. s.	n. s.
Bar.	2.90	0.74	n. s.	n. s.	1 > 3,4 *	1 > 2 *	n. s.	n. s.	n. s.
SE	3.65	0.78	n. s.	n. s.	n. s.	n. s.	5 > 1,3 **	n. s.	2,3 > 5;3 > 1 ***
Int.	3.97	0.75	n. s.	n. s.	4 > 2 **	n. s.	5 > 1,3 **	n. s.	n. s.
Know.	3.72	0.86	n. s.	n. s.	n. s.	2,3 > 1 ***	5 > 1,2,3 ***	4 > 1,2,3,5 ***	5 > 3,4 ***
Att.	3.58	0.89	n. s.	n. s.	n. s.	2,3 > 1 ***	5 > 1,2,3 ***	4 > 1,2,3,5 ***	5 > 3,4;1 > 3 ***

**Table 4:** Associations of variables.

	M1					M2				
	Unstd.		Std.	t	*p*	Unstd.		Std.	t	*p*
	B est.	SE	β							
(constant)	3.136	0.302		10.388 ***	0.000	0.994	0.318		3.127 **	0.002
Gender	0.051	0.089	0.033	0.566	0.572	0.026	0.072	0.017	0.365	0.716
Age	0.043	0.039	0.065	1.102	0.271	0.023	0.032	0.035	0.717	0.474
Marriage	0.083	0.091	0.053	0.909	0.364	0.075	0.073	0.048	1.027	0.305
Income	0.106	0.037	0.175	2.885 **	0.004	0.022	0.030	0.037	0.742	0.458
Education.	0.028	0.076	0.022	0.370	0.711	0.034	0.061	0.026	0.548	0.584
Occupation	0.016	0.024	0.035	0.670	0.503	−0.014	0.019	−0.030	−0.724	0.470
District	0.034	0.030	0.066	1.164	0.245	0.031	0.025	0.059	1.237	0.217
Threat						0.226	0.051	0.189	4.401 ***	0.000
Net benefit						0.206	0.032	0.275	6.410 ***	0.000
Self-efficacy						0.428	0.042	0.441	10.084 ***	0.000
R	0.198					0.625				
R^2^	0.039					0.390				
Adj. R^2^	0.021					0.373				
△R^2^						0.351				
F						23.278				
p						0.000				

*n* = 375, ** *p* < 0.01, *** *p* < 0.001.

**Table 5:** Regressions of major constructs on district.

DV	Perceived Threat		Likelihood of Action		PSU Intention	
District	*Β*	*p*	*β*	*p*	*β*	*p*
A1 Cianjhen	−0.257 ***	0.000	−0.128 *	0.041	−0.131 *	0.039
A2 Daliao	−0.262 ***	0.000	0.069	0.240	0.034	0.565
A3 Fongshan	−0.301 ***	0.000	−0.018	0.759	0.029	0.633
A4 Linyuan	−0.307 ***	0.000	−0.012	0.836	0.023	0.691
A5 Siaogang	0.000		0.000		0.000	
R	0.359		0.264		0.233	
R^2^	0.129		0.070		0.054	
Adj. R^2^	0.105		0.044		0.029	
F	5.401 ***	0.000	2.720 **	0.003	2.098 *	0.024

DV, Dependent variables; * *p* < 0.05; ** *p* < 0.01; *** *p* < 0.001.

**Table 6:** Moderating effects of knowledge and attitude.

IV-DV	Unstd.		Std.	t	*P*
Threat	B est.	SE	β		
(constant)	0.039	0.275		0.140	0.889
Threat ^(Z)^	0.237	0.060	0.237 ***	3.962	0.000
KA ^(Z)^	0.004	0.073	0.003	0.057	0.954
Threat ^(Z)^ x KA ^(Z)^	−0.100	0.049	−0.103 *	−2.038	0.042
Net benefit					
(constant)	−0.502	0.222		−2.255 *	0.025
Likelihood ^(Z)^	0.369	0.048	0.369	7.719 ***	0.000
KA ^(Z)^	0.137	0.060	0.111	2.297 *	0.022
Net benefit ^(Z)^ x KA ^(Z)^	0.021	0.046	0.022	0.450	0.653
Self-efficacy					
(constant)	−0.687	0.204		−3.369 **	0.001
Self-efficacy ^(Z)^	0.517	0.044	0.517	11.665 ***	0.000
KA ^(Z)^	0.188	0.055	0.152	3.456 **	0.001
Self-efficacy ^(Z)^ x KA ^(Z)^	0.048	0.046	0.046	1.038	0.300

*n* = 375; ^(Z)^, Z score, R^2^, R-squared; adj. R*^2^*, adjusted R^2^; F, F statistic value; * *p* < 0.05; ** *p* < 0.01; *** *p* < 0.001.
